# Parental Overprotection and Locus of Control as the Mechanisms Explaining the Relationship Between Parent and Child Anxiety: A Multiple Mediation Model

**DOI:** 10.1007/s10578-024-01757-4

**Published:** 2024-09-12

**Authors:** Yosi Yaffe

**Affiliations:** Department of Special Education, Tel-Hai Academic College, 12208 Kiryat Shmona, Upper Galilee, Israel

**Keywords:** Parent anxiety, Child anxiety, Parental locus of control, Overprotection

## Abstract

The study probes the role played by parenting control practices and parental locus of control in the relationship between parent and child anxiety. The study particularly aims at probing these matters in light of the parental gender-specific role, striving to improve our understanding of the differential etiological contribution of mothers’ and fathers’ anxiety and parental practices to child’s anxiety. The study consisted of 316 parents (159 mothers and 157 fathers) who reported their own and their child’s anxiety using valid instruments. The general path model used in the study exhibited an adequate fit to the data, generally confirming our theory regarding the direct and indirect associations between parent–child anxiety. Using SEM multiple group analysis for parental gender, a strong-direct unique association was found between parent and child anxiety. For mothers, this association was partially mediated by maternal overprotection. Finally, maternal external locus of control was positively associated with child anxiety, after accounting for the effects of all other maternal variables. The study’s findings and limitations are profoundly discussed in light of parental gender differences.

Anxiety disorders are among the most common mental health conditions in children and adolescents [1]. The prevalence of anxiety disorders in children and adolescents can vary by age and gender, with some estimates suggesting that between 15 and 20% of young people will experience an anxiety disorder before reaching adulthood [2–4]. Other epidemiological estimations suggest even higher rates of anxiety disorder of any kind (about 32%), with about 8.3% causing severe impairment [5]. Youngsters with disabilities seem to be at even greater risk of experiencing anxiety and developing anxiety disorders [6]. Given the detrimental consequences of childhood anxiety disorders on educational and social well-being, and even physical health, in both the short and long term [7], it is essential to continuously investigate the phenomenon’s etiology, especially in the familial contexts of parental child-rearing styles and behaviors.

## Prominent Links Between Parenting and Children’s Anxiety

The way parents raise their children, including their parenting style and specific behaviors, shapes the environment and the climate in which children grow and influences their psychological development. An extensive body of research has provided valuable insights into the connection between parenting and children’s anxiety (refer to [8], and [9]). In general, it has dealt with how different patterns of low levels of parental acceptance/warmth and excessive control exercised by both parents are related to children’s anxiety. When we closely examine the collective findings from various studies as reflected in at least three meta-analytic works, it becomes apparent that there is stronger evidence linking parental controlling behaviors to children’s anxiety disorders, as opposed to parental acceptance-rejection behaviors (refer to [10], and [11]). Of these patterns of parenting styles and practices, parental overcontrolling behaviors such as lack of autonomy granting seem more closely and more distinctly related to childhood anxiety. Excessive parental control is often characterized by the use of strict disciplinary measures, excessive regulation of the child’s actions, overprotectiveness, and the exertion of psychological control, such as instructing them on what to think and feel [12, 13]. These behaviors are in direct contrast to promoting a sense of autonomy in the child. Since early childhood, overcontrolling parenting tends to precede and interfere with the child’s opportunities to self-regulate his or her emotions and behaviors, resulting with diminishing the child’s experiences of practicing emotional regularity [14], especially with regard to situations of fear arousal. Indeed, in two of the most comprehensive meta-analysis works conducted in the past decades, parental overcontrol was found to have a medium to strong association with children’s anxiety (in overrepresentation of boys samples the effect size was *d* = 0.52, whereas the overall effect size for samples containing overrepresentation of girls was *d* = 0.63,[15]), indicating that anxious children and adolescents are very likely to be raised by overcontrolling parents [9]. In particular, parental lack of autonomy granting was identified as the most predictive parental controlling behavior in the context of children’s anxiety, explaining an average of 18% of the variance in the latter variable in a meta-analytic inspection of 47 studies [10].

In both meta-analyses, parental overcontrolling behaviors, especially autonomy granting, demonstrated a significantly stronger association with childhood anxiety than parental acceptance-rejection behaviors such as warmth, which led the researches to conclude that excessive parental control can play a unique role in relation to anxiety disorders in children and adolescents, whether by serving as the primary cause of anxiety, a reaction by parents to their child’s anxiety, or an outward manifestation of the parents’ own anxious tendencies [10, 11]. Accordingly, the use of broad parenting dimensional variables in the etiological research of childhood anxiety, which included parental warmth/acceptance variables, might have underestimated the potential strength of the parent–child anxiety relationship [10, 15], resulting in a relatively small overall effect size in several meta-view studies [10, 16]. Indeed, several recent review works focusing on this very issue have shown that the evidence linking between the parental acceptance-rejection dimension and childhood anxiety is far less etiologically compelling in comparison to that referring to parental control [9, 17, 18]. Hence, future etiological research investigating the parental role in children’s and adolescents’ anxiety may benefit from focusing on overcontrolling parenting behaviors rather than on the parental acceptance-rejection dimension. Such a distinct overcontrolling parental behavior is overprotection, which has been associated consistently and specifically in the research literature with children’s anxiety [19–23]. In contrast to autonomy granting, parental overprotection can be characterized as parenting behaviors that are excessively supportive and nurturing, thereby restricting children’s chances to deal autonomously with age-appropriate challenges [22, 24]. While autonomy granting and overprotection intuitively and theoretically constitute contrasting parental rearing behaviors, some theoretical and empirical evidence suggest that these parental control patterns are not overlapping opposite constructs (i.e., opposite ends of the same construct), but can be considered as a closely related, distinct constructs [25–28]. Especially in the context of children’s anxiety, these and other dimensional and sub-dimensional parental control constructs might play a distinct and differential etiological role [10, 21]. In this regard, [29] referred to the prevention of the child’s autonomy and parental overcontrolling behaviors as a merely two single aspects of parental overprotection related to the child’s anxiety. In fact, overly rigid and strict parental behaviors, typically expressed in over overcontrolling parenting, such as the authoritarian style [30], are not completely in line with parental overprotection, which, according to some theoretical and clinical views, involves expressions of parental warmth [22, 26, 31, 32]. Yet many studies did not sufficiently discern, and simultaneously use, parental control variables such as overprotection, overcontrol, and autonomy granting as separate measurements of distinct constructs. Hence, particularly in the context of children’s anxiety, challenging the premise that parental lack of autonomy granting or overcontrol equal overprotection per se might be beneficial to our understanding of the parental etiological role.

## The Relationship Between Parent and Child Anxiety

The body of work dealing with the connection between parents’ and children’s anxiety may help to clarify the mechanism underlying the relationships between parenting practices and behaviors and children’s anxiety. These research data provide ample evidence for the positive association between parent and child anxiety [33–37], including specific anxiety symptoms [38, 39], generally suggesting that anxious children and adolescents are considerably more likely to be raised by anxious parents. Beyond the etiological attribution of children’s anxiety to shared familial genetic vulnerabilities [40], the literature points out several theoretical pathways in which anxiety is transmitted between parent and child through environmental influences, part of which could be related to parents’ and/or children’s anxious characteristics. Parental anxiety was suggested to evoke ‘anxiety-enhancing’ parenting behaviors [15], such as modeling a fearful cognitive style and exerting overprotective/overcontrolling behaviors, partly exhibited by reinforcing anxious cognition and avoidant behavior and transmitting an increased appraisal of threat/risk through discussion [41–44] (For a more comprehensive review of the theoretical mechanism of transferring anxiety between parents and children in the family, see [11] and [43]). Indeed, in a recent systematic review encompassing 13 research articles, parental anxiety was found to be significantly associated in the research literature with mothers’ overprotective behaviors [22], although the overall magnitude of the parental effect was medium at most. Alternatively, in an earlier a meta-analytic review focusing on the relationship between child and parent anxiety and parental control, solely the child’s anxiety was associated, strongly, with parental control (*d* = 0.58). However, the association of parental anxiety with parental control in this meta-analysis was generally insignificant, apart from a small significant effect in a group of school-aged children [15]. Given the most prominent role played by parental control in the etiology of children’s anxiety, it is essential to probe further the parenting mechanism determining parental control and linking it with children’s anxiety. This goal may be better achieved, as said earlier, by simultaneously incorporating several variables of parental control (e.g., autonomy granting, psychological control, overprotection, etc.), which will enable the inspection of their specific interplay with parental anxiety and would elucidate their unique and distinct contribution to predicting children’s anxiety. Indeed, the majority of studies addressing the relationship between parental rearing practices within the framework of a child’s anxiety have primarily focused on either the child’s anxiety or the parental anxiety, while few studies have assessed parental rearing practices alongside both child anxiety and parental anxiety [19]. Moreover, there is some research evidence suggesting that parental control, especially overprotection, may serve as the mechanism partially explaining the relationship between parent–child anxiety. In a study conducted by [45] with preschoolers and their mothers, the researchers found an indirect association between maternal trait anxiety and the child’s separation anxiety, which was mediated by the mother’s overprotective practices. In this study, mothers with greater anxiety demonstrated higher usages of overprotective practices, which, in turn, explained the child’s anxiety. Indeed, in Parker’s earlier studies, parental trait anxiety was the best predictor of overprotection [45]. Similar findings were observed in a longitudinal study with school age children that investigated the mechanism explaining the association between parent and child anxiety [46]. Accordingly, maternal (but not paternal, since paternal anxiety was not related to overcontrol) overcontrol acted as a significant mediator of the relationship between maternal anxiety obtained in the study’s data collection at time one (T1) and the child’s anxiety at time two (T2). In this regard, maternal overcontrol partially explained the increase in children’s anxiety over time in positive relation with maternal anxiety. Both pieces of evidence suggest that parental overcontrolling behaviors in child-rearing could result from, or be related to, parental anxiety, which, in turn, at least partially explains the child’s elevated anxiety.

## Attributions of Control in Relation to Parent and Child Anxiety

The degree to which events tend to elicit feelings of anxiety or negative emotions relies, to a great extent, on the level of control the organism has over those events, suggesting that a sense of control can play a central role in reducing or inducing such emotional reactions [24]. In this sense, Barlow [47] referred to (lack of) control as the core of anxiety and to low perceived control as the diathesis for anxiety disorder. Thus, early exposure to uncontrollable events, predominantly experienced in various contexts of parent–child relations, can be seen as a key avenue in the formation of anxiety disorders, as such experiences may promote an increased propensity to interpret events as not subject to one’s regulation [11, 24]. The inclination to attribute control over events either to external or internal causes can be referred as locus of control [48]. Locus of control is a psychological trait referring to individuals’ belief regarding the extent to which they can control events and outcomes in their life, and, according to [48], can be sorted into external and internal locus of control types. Locus of control is an essential concept in the field of personality psychology and has implications for various aspects of an individual’s life, including their behavior, motivation, decision-making, and especially mental well-being. As for the latter aspect, ample research data show that external locus of control, as opposed to internal locus of control, is associated with poor mental health and psychological well-being both among parents and children/adolescents with and without disabilities. This includes the child’s externalizing and internalizing problems, anxiety and depression, parental stress, and more (e.g., [23, 49–54]). Moreover, in several research studies, the child’s locus of control was even found to serve as the mediating mechanism that at least partially explains the association between parental variables (such as insecure attachment and overprotection) and the child’s mental health problems, especially internalizing problems and anxiety [23, 55]. Surprisingly, however, much less empirical information can be found in the research literature about the potential influence of parental locus of control on the child’s mental health and emotional well-being, especially given the correspondence between the parent’s and the child’s attributions and the similarity between the parent’s and the child’s locus of control over time [56, 57]. Given the importance of parental control and, in turn, control perceptions in the development and maintenance of anxiety in children and adolescents [11, 24], it could be beneficial to develop a better understanding regarding the possible role of locus of control, specifically parental locus of control, in this regard. Parental locus of control (PLOC) pertains to how parents perceive their ability, competence, and effectiveness in their role as caregivers. It holds significance within the realm of parenting, as it is closely linked to how parents view the origins of their children’s challenges and problems [58]. Hence, parental locus of control can be seen as the manifestation or reflection of the parent’s general of non-specific locus of control in the situation-specific context of child-rearing [52]. Parents who possess an external locus of control are inclined to attribute their children’s development and behavior to external forces beyond their influence [50]. Numerous research studies reveal promising evidence regarding the predictivity of parental locus of control of the child’s psychological, social-educational [50, 57], and even physical well-being, part of which also provides support for the causal role of that parental variable in these contexts. For example, in a recent longitudinal study, parental external locus of control measured during pregnancy, and the child’s external locus of control measured at the age of 8 years, were found to be associated with the child’s obesity later in adolescence [59]. Another longitudinal study tested how changes in parental locus control over a 6-year span were associated with differences in elementary school children’s behaviour as rated by their teachers [57]. The findings affirmed the notion that parental locus of control might be influential on children’s personal and social adjustment, as the study’s parents who remained or became external in locus of control were found to have children with more behavior difficulties than those parents who remained or became internal in locus of control. Despite this compelling evidence, there is a serious shortage of studies investigating the possible linkages between parental locus of control and children’s psychological outcome variables, especially anxiety disorders. In this regard, we found only one old study that specifically investigated and demonstrated the positive relationship between parental external locus of control and children’s anxiety, for both mothers and fathers [60]. The necessity of expanding this course of research by focusing on the parent’s locus of control is essential in promoting the knowledge about the familial etiology of children’s anxiety, especially considering this variable within the wider context or framework of parental controlling behaviors and practices.

## The Current Study

**Fig. 1:**
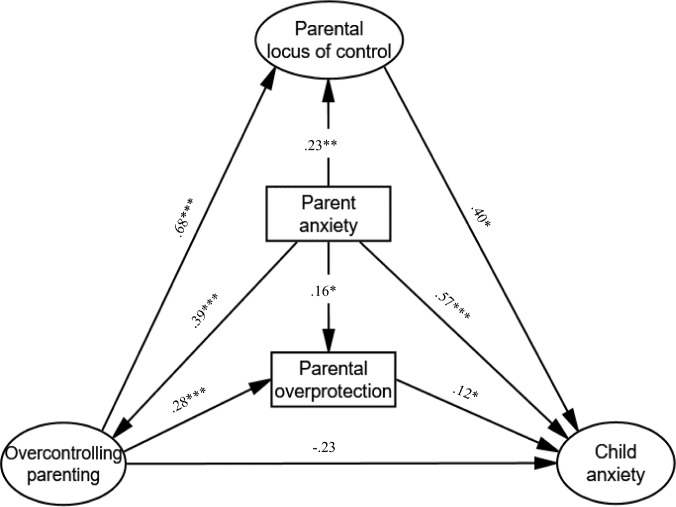
Model of direct and indirect effects for the general sample. Model fit: CFI = 0.93, RMSEA = 0.07, GFI = 0.95, SRMR = 0.05. Indices represent standardized estimates. *p ≤ .05, **p ≤ .005, ***p ≤ .001. Significant Indirect Effects on Child Anxiety. Parent anxiety (via locus of control): *β* = 0.09, *p* = 0.03; Overcontrolling parenting (via parental overprotection): *β* = 0.03, *p* = 0.05; Overcontrolling parenting (via locus of control): *β* = 0.28, *p* = 0.02

In light of the above literature, the current study sought to probe the relationship between parent and child anxiety, while accounting for the possible mechanism played by parental overcontrolling variables and parental locus of control in this context. Specifically, the study aims to test a theoretical model that integrates numerous parental control variables (i.e., autonomy granting, overcontrol, and overprotection), along with parental locus of control, as the mechanisms explaining the relationship between parents’ and children’s anxiety. Given the importance of control variables in the etiology of child’s anxiety, the current study will strive to identify the unique predictive role played by these parental variables in this context. Using a cross-sectional design, the study sought to further expand upon the limited studies testing parental rearing practices alongside both child anxiety and parental anxiety [19], which have not been significantly changed over the years in this regard. Given that mothers’ and fathers’ parenting practices may very [61], it is essential to consider gender differences when investigating the associations between parent and child anxiety and parental control [15, 62]. Therefore, we will initially consider the fit of the proposed research model in the general sample, and then test the research hypotheses separately for mothers and fathers. Based on the review of the literature above, the following specific hypotheses are addressed as part of an integrative path model embodying the variables’ direct and indirect effects (Fig. 1):

H1: Parent–child anxiety would be positively and directly associated.

H2: Parent anxiety would be positively associated with the parental control variables of overprotection, external locus of control, and overcontrolling parenting.

H3: Parent–child anxiety would be associated indirectly via parental overprotection and parental external locus of control as mediators.

H4: Overcontrolling parenting would be associated with child anxiety both directly and indirectly via parental overprotection and external locus of control.

## Method

### Participants and Procedure

The participants were 316 parents (159 mothers and 157 fathers) whose age ranged from 30 to 58 (*M*age = 44.07, SD = 5.08). The parents had at least one early adolescent children (44% girls), whose age ranged between 10 and 14, regarding whom they reported their parenting styles and behaviors, and the child’s anxiety level. The majority of parents were married (88%), and the rest either divorced or single. The sample’s family size was of 4.96 ± 1.46 individuals in average. The majority of the sample was secular Israeli Jewish parents (53.8%), while the rest of the parents defined themselves as either religiously traditional Jews (29.7%) or Israeli religious Jewish (16.4%). The study utilized an anonymous battery of questionnaires in Hebrew, which were administered online by an Israeli professional survey provider. Recruited participants received a link to an online research portal, where they could access the study-related information and to read their rights. Informed consent was obtained digitally from participants, affirming their willingness to participate after understanding the provided information. Participants had the option to withdraw from the study at any point, or to contact the researcher’s representatives in any case they required further details or encountered unforeseen issues or distress. The research procedure was approved by the institutional review board (IRB) (Ref. number 21-6/2023) of Tel-Hai college as part of a broader study on parent–child relations.

### Instruments

#### Parental Locus of Control (PLOC; [58])

The original instrument contains 47 items, generally designed to measure the parental perceptions of control, efficacy, and competence in parenting a child [63]. In the current study, we used the revised short form of the parental locus of control scale (PLOC-SFR; [51]), which enables to overcome numerous flaws in the original 5-scale instrument [51] and its usage is growing popular in recent years [63]. The short-form PLOC includes 24 items based on four parental domain scales: *parenting self-efficacy* (e.g., “What I do has little effect on my child’s behavior”); *parental responsibility* (e.g., “There is no such thing as good or bad children- just good or bad parents”); *child control of parents’ life* (e.g., “I feel like what happens in my life is mostly determined by my child”); and *parental control of child’s behavior* (e.g., “My child’s behavior is sometimes more than I can handle”). Parental responses are measured on a 5-point Likert scale, ranging from 1 (strongly disagree) to 5 (strongly agree), with higher scores representing more external locus of control. The internal consistency reliability indexes recorded for the instrument’s scales in the current sample were acceptable (α = 0.67–0.76) (considering the low number of items included in each scale), apart from the *responsibility* scale, whose coefficient level approached 0.60.

#### Parental Overprotection (OP; [32])

The Parental Overprotection Measure was employed to assess parental self-reported overprotective behaviour. This tool comprises 19 items designed to evaluate parenting actions that limit a child’s exposure to perceived threats or potential harm. The items primarily focus on specific behaviors or situations rather than general attitudes or beliefs, such as questions inquiring about actions like keeping a child close while playing or shielding them from criticism. Parents are asked to rate how closely each item reflects their typical response on a 5-point scale, ranging from 0 (not at all) to 4 (very much). Higher scores on this scale reflect higher level of parental overprotection. Previous research has established the OP measure’s reliability and validity, demonstrating high internal consistency (Cronbach’s alpha = 0.87) and test–retest reliability [32]. Additionally, it has shown good construct and predictive validity when utilized with community samples of parents with children aged 7 to 12 years [20]. In the current study, the Cronbach’s alpha coefficient for the OP scale was 0.90 and 0.93 (for mothers and fathers, respectively), indicating an excellent internal consistency reliability index. The scores recorded for the scales in the current study are reported in Table 1.

**Table 1:** Descriptive statistics and zero-order correlations of variables of interest by parental gender

	1	2	3	4	5	6	Male *M* (SD)	Female *M* (SD)	*t*	Cohen’s *d*
1. Autonomy granting	–	−0.39**	−0.28**	−0.28**	−0.34**	−0.19*	3.75 (.61)	3.63 (.84)	2.19*	.25
2. Parental overcontrol	−0.48**	–	0.39**	0.44**	0.33**	0.30**	19.81 (5.84)	18.33 (5.43)	2.33*	.26
3. Parent anxiety	−0.14	0.18*	–	0.39**	0.29**	0.61**	3.71 (3.92)	3.54 (3.05)	.49	.05
4. Parental locus of control	−0.31***	0.37**	0.27**	–	0.21*	0.28**	2.37 (.35)	2.34 (.39)	.88	.10
5. Parental overprotection	−0.22*	0.06	0.23*	0.16*	–	0.24*	32.59 (13.77)	30.6 (11.7)	1.16	.13
6. Child anxiety	−0.07	0.17*	0.60**	0.37**	0.33**	–	7.37 (3.44)	8.00 (3.88)	1.53	.17
Mean (general sample)	3.69	19.06	3.63	2.36	31.75	7.64	–	–	–	–
SD (general sample)	0.47	5.68	3.51	0.37	12.79	3.65	–	–	–	–

Figures above the diagonal represent the father’s data and figures below the diagonal represent the mother’s data

*p ≤ .05, **p ≤ .001

#### Overcontrolling Parenting

In the current study, overcontrolling parenting was measured using two distinct scales from two valid questionnaires. To gauge the parental overcontrol practice, we used the authoritarian parenting scale taken from the Parenting Styles and Dimensions Questionnaire (PSDQ; [64]). Authoritarian parental control is described as a distinct form of a strict and excessive parental control, often characterized by threats, fear and guilt induction, coercion, love withdrawal, intrusiveness, and punishment [12, 30, 65, 66]. In the current study, we used the 12-item scale authoritarian control (“I scold or criticize when our child’s behaviour doesn’t meet our expectations”), excluding 3 items referring to corporal punishment (which is a legally banned parental practice in the country where the study took place). The authoritarian scale encompasses three parental overcontrol sub-scales, including Physical Coercion, Non-Reasoning/Punitive, and verbal hostility. In the current sample, we obtained an identical good index of internal consistency reliability for mother and fathers (α = 0.85), which is consistent with previous evidence about the tool’s reliability both in its original and Hebrew form (PSDQ; [64, 67]).

While the PSDQ is an empirically evidence-based valid and reliable instrument for assessing parenting styles [68], its authoritarian scale fails to reflect distinctly the parental overcontrol eminent aspect of denying the child autonomy. Therefore, for the purposes of assessing overcontrolling parenting, in the current study we also used the parental report form of the autonomy scale taken from the Parenting Style Inventory II (PSI-II,[69, 70]), which is a 5-item scale measuring aspects of parental autonomy granting with adolescent children (e.g., “I give my child a lot of freedom”; “I tell my child that my ideas are correct and that s/he shouldn’t question them” – a reversed item; “I believe my child has a right to have his/her own point of view). The researchers have reported a moderate Cronbach’s Alpha index of reliability for the autonomy scale (α = 0.75), while in the current sample we recorded an inadequate internal consistency reliability index (α = 0.60), which could not be improved by omitting items from the scale, partially due to the small number of items composing the scale. The scores recorded for these two scales in the current study are reported in Table 1.

#### The Generalized Anxiety Disorder 7-Item Scale (GAD-7; [71])

Parent’s general anxiety was assessed using the 7-item scale, a self-report measure designed primarily as a screening and severity measure for generalized anxiety disorder in adult individuals. The response for an item is given on a 5-point scale (“not at all”, “several days”, “more than half the days”, and “nearly every day”, respectively). The scale’s total score ranges from 0 to 21, with a higher score representing a higher level of anxiety. The scores recorded for the scale in the current study are reported in Table 1. In the current study, the Cronbach’s alpha coefficient for the OP scale was 0.82 and 0.88 (for females and males, respectively), indicating a good internal consistency reliability index.

#### Spence Children’s Anxiety Scale – Brief Version (SCAS-P-8; [72])

The SCAS-P-8 is a brief version of the original children’s anxiety questionnaire that comprised of 38 items, intended for the evaluation of a child’s anxiety symptoms. It consists of child report (SCAS-C; [29, 73]) and parent report (SCAS-P [74],) versions, the latter of which was used in the current study. The items within the brief version address symptoms related to anxiety disorders as outlined in DSM–IV, including separation anxiety, generalized anxiety, social phobia, and panic/agoraphobia. Respondents rate these items on a 4-point scale (ranging from 0 to 3, representing "never" to "always"), and the total score is the sum of responses to all 38 items. The scores recorded for the scale in the current study are reported in Table 1. In the current study, the Cronbach’s alpha coefficient for the general SCAS-P-8 scale was adequate given the scale’s number of items (α = 0.78), which is consistent with reliability data reported for the original brief version of the SCAS.

### Data Analysis

Data were received from the survey provider without missing values, and therefore no missing data handling procedures were required. To improve model parsimony, a single measurement model was established while constructing latent variables for overcontrolling parenting (composed of the PAQ authoritarian scale and the PSI-II autonomy granting scale as indicator measures), parental locus of control using four scale indicators (i.e., efficacy, responsibility, child control, parent control), and child anxiety using 3 scale indicator measures (separation, social, and generalized). After removing the responsibility scale from the locus of control variable (due to poor factor loading), all factor loadings were > 0.54 and the model fit the data well (see Fig. 1). Thus, regarding overcontrolling parenting and parental locus of control, the corresponding latent variables represent high overcontrolling parenting (with autonomy granting negatively loaded on this factor) and external locus of control perceptions (respectively). Regarding child anxiety, the corresponding latent variable represents a high anxiety level. Other variables included in the model were used as observed variables (i.e., parental overprotection and parent anxiety), as they contain a single scale variable. Descriptive statistics and correlational analyses were conducted using the IBM-SPSS 28 package. Figure 1 displays the research model embodying the standardized direct and indirect effects, which were assessed through path analysis in AMOS version 28.0. Measurement invariance across parental gender for the proposed research model was initially determined using SEM multigroup analysis. Significant effects were compared across gender using multiple-group analysis and pairwise parameter comparisons, which produces a Z score indicating significant differences. The significance of indirect effects was scrutinized using 5000 bootstrap samples, accompanied by 95% bias-corrected confidence intervals. These direct and indirect effects were subsequently utilized for hypothesis testing separately for mothers and fathers.

## Results

### Correlation and Mean Comparison

Table 1 presents the descriptive statistics and intercorrelations for the research variables by parental gender. As can be seen, all the parental variables were significantly, positively correlated in the fathers’ sample, which principally corresponds to the general sample. Relative to the fathers, in the mothers’ sample a few correlational exceptions were observed, as detailed below. For both parents, the most prominent association among the research variables was between parent’s and child’s anxiety, whose direction is positive, as expected. Moreover, all maladaptive parental variables (such as parental external locus of control, parental overprotection, and parental overcontrol) were moderately related to higher child anxiety. Paternal autonomy granting was negatively associated with all the parental variables, while also moderately related to the child’s anxiety. Maternal autonomy granting, however, was statistically associated only with maternal overcontrol and external locus of control, while unrelated to either the parent’s or the child’s anxiety. Regarding both parents, parental autonomy granting was merely weakly to moderately (though significantly), reversely correlated with overprotection, indicating conceptual discrimination between the two constructs. Ultimately, unlike fathers, maternal overcontrol exhibited a weak (yet significant) statistical association with the parent’s and the child’s anxiety, and was not related to parental overprotection.

Upon testing parental gender effects on the study variables, mean differences were identified only for autonomy granting (mothers outscoring fathers) and overcontrol (fathers outscoring mothers). In this table, gender differences in regard to child anxiety were also considered (i.e., in terms of the child’s gender regardless of parental gender), yielding similar anxiety levels between boys and girls according to the parental reports. Likewise, the child’s gender differences in the parental variables was found merely for fathers regarding a single variable, exhibiting slightly, yet significantly, higher levels of parental overcontrol toward boys compared to girls (*t*(155) = 2.274, *p* = 0.024). Finally, the child’s and parent’s age were not statistically associated with either of the study variables and were therefore excluded from Table 1.

### Introducing the Research Model in the General Sample

The research hypotheses are embodied in an integrative path model, which is presented in Fig. 1, which describes the direct and indirect associations between the research variables. In this section, the model is introduced for the sample as a whole, in order to initially characterize the general unique associations between the variables of interest as part of the proposed model. Later, we test the research hypotheses using the current model separately for mothers and fathers, based on the primary analysis showing partially differential correlational patterns across parental gender.

#### Significant Indirect Effects on Child Anxiety

The model displayed in Fig. 1 exhibited acceptable fit indices according to Kline’s [75] and Hooper et al. [76] recommendations of goodness-of-fit thresholds, despite its significant chi-square value (*χ2*(28) = 79.01, *p* < 0.01; *χ2*/*df* = 2.82), which is an index sensitive to a sample size. According to the model’s findings, the parental variables of locus of control, parental anxiety, and overprotection all significantly predict the child’s anxiety in the general sample, which is generally consistent with our hypotheses regarding the model’s direct effects. Contrary to our hypothesis, however, overcontrolling parenting (which is composed of two indicators: parental autonomy granting and parental overcontrol, with the former observed variable negatively loaded on this factor) was not directly associated with child anxiety. Rather, overcontrolling parenting was positively associated with the child’s anxiety indirectly through both parental overprotection and external locus of control (given that the direct, positive associations with these variables were significant), indicating that these maladaptive parenting practices are associated with elevated child anxiety due to higher levels of those mediating variables among parents. Likewise, parent anxiety was related to higher child anxiety indirectly through locus of control (with which the direct, positive association is significant), meaning that apart from the direct link between these variables, the association between parent–child anxiety in the general sample can be partially explained by higher external parental locus of control. This was approximately true also with respect to parental overprotection as a mediating variable of the relationship between parent–child anxiety, with the indirect prediction weight of its path in this model approaching significance (*β* = 0.02, *p* = 0.07).

Taken together, the parental variables explained approximately 58% of the variance of the child’s anxiety scores.

### Hypotheses Testing Among Mothers and Fathers

Based on the model presented in Fig. 1, in the current section we test the specific research hypotheses separately for fathers and mothers. The statistical results from the path model using structural equation modeling appear in Table 2, elaborating the regression weights for the model’s direct and indirect effects and their CI values by parental gender. A SEM multigroup analysis was used to determine measurement equality of the model variables between mothers and fathers (by comparing the unconstrained model to the model constraining the factors loadings), which indicated a configural and metric measurement invariance for the model across parental gender (CMIN-*χ2*(6) = 11.30, *p* = 0.08).

**Table 2:** Path estimate (SE) and Bootstrapped 95% CIs for the research model’s direct and indirect effects across parental gender (mothers/fathers)

	Unstandardized (B)			Standardized (β)
	Estimate (SE)	Lower 95% CI	Upper 95% CI	Estimate (SE)
Direct effect				
Parent anxiety–Overcontrolling	0.31 (0.13)/0.56 (0.14)	0.05/0.33	0.68/0.88	0.22 (0.10)/0.52 (0.10)
Overcontrolling-Overprotection	0.28 (0.26)/1.40 (0.51)	−0.32/0.55	1.40/2.57	0.10 (0.13)/0.45 (0.13)
Overcontrolling-Locus of control	0.04 (0.01)/0.05 (0.02)	0.01/0.03	0.08/0.09	0.62 (0.18)/0.72 (0.13)
Parent anxiety-Over protection	0.79 (0.31)/0.19 (0.41)	0.14/−0.59	1.47/1.06	0.21 (0.08)/0.06 (0.12)
Parent anxiety-Locus of control	0.02 (0.01)/0.01 (0.01)	0.02/−0.01	0.04/0.03	0.27 (0.16)/0.13 (0.17)
Locus of control-Child anxiety	1.78 (2.64)/0.36 (2.04)	0.50/−1.86	6.89/4.40	0.38 (0.57)/0.10 (0.52)
Overprotection-Child anxiety	0.03 (0.01)/0.003 (0.01)	0.01/−0.02	0.05/0.02	0.24 (0.10)/0.03 (0.11)
Parent anxiety-Child anxiety	0.23 (0.04)/0.19 (0.05)	0.07/0.11	0.34/0.28	0.57 (0.17)/0.63 (0.13)
Overcontrolling-Child anxiety	−0.05 (0.05)/−001 (0.15)	−0.33/−0.26	0.06/0.22	−0.17 (0.52)/−0.004 (0.53)
Indirect effect (total)				
Parent anxiety-Overprotection	0.09 (0.13)/0.82 (0.32)	−0.09/0.34	0.46/1.60	0.02 (0.03)/0.23 (0.09)
Parent anxiety-Locus of control	0.01 (0.01)/0.03 (0.01)	0.002/0.01	0.05/0.06	0.14 (0.08)/0.37 (0.12)
Overcontrolling-Child anxiety	0.07 (0.16)/0.02 (0.14)	−0.001/−0.11	0.44/0.30	0.26 (0.51)/0.08 (0.48)
Parent anxiety-Child anxiety	0.07 (0.07)/0.02 (0.03)	0.01/−0.08	0.25/0.08	0.14 (0.17)/0.06 (0.11)

*CI* confidence interval, *SE* standard error

Hypothesis 1 stated that parent–child anxiety would be positively and directly associated. As can be seen in Table 2, this hypothesis was supported both for mothers (*β* = 0.57, *p* < . 001) and for fathers (*β* = 0.63, *p* < . 001), without significant gender variation (*Z* = 0.631, *p* = 0.53), which indicates that anxiety in parents is a strong, unique predictor of their child’s anxiety according to parents’ reports.

Hypothesis 2 stated that parent anxiety would be positively associated with the parental variables of overprotection, external locus of control, and overcontrolling parenting. Among mothers, this hypothesis was fully supported (as indicated by the positive CI values obtained for each link in Table 2), while among fathers, the hypothesis was supported solely regarding overcontrolling parenting as a dependent variable (parental gender differences in the associations between parent anxiety and overcontrolling parenting were insignificant,* Z* = 1.591, *p* = 0.11). Additionally, the latter also significantly mediated the positive association between parent anxiety and parental external locus of control, partially for mothers (*β* = 0.14, *p* = 0.01) and fully for fathers (*β* = 0.37, *p* < . 001).

Hypothesis 3 stated that parent–child anxiety would be associated indirectly via parental overprotection and parental external locus of control as mediators. For mothers, this hypothesis was partially supported by the findings. As can be seen in Table 2, the direct paths between mothers’ anxiety and the mediators and between the mediators and child anxiety were all significant (the precondition for mediation of the effect is confirmed). As a result of this, the total indirect effect for mother–child anxiety was significant (*β* = 0.14, *p* = 0.02). However, the bootstrap analysis of the individual indirect effects between mother–child anxiety yielded significant CI values (i.e., positive range, excluding zero) only via maternal overprotection as a mediator (*β* = 0.05, *p* = 0.01), but not via maternal locus of control (*β* = 0.10, *p* = 0.10). Taken together, the direct and indirect links between parent–child anxiety indicate that anxiety in mothers is associated with child’s anxiety partially due to mothers’ overprotection. With respect to fathers, however, this hypothesis was not supported, as the direct links between parent’s anxiety and the mediators (i.e., parental overprotection and locus of control) and between the mediators and the child’s anxiety were insignificant, resulting in an insignificant total indirect association between the father and the child’s anxiety.

Hypothesis 4 stated that overcontrolling parenting would be associated with child anxiety both directly and indirectly via parental overprotection and external locus of control. In terms of a direct effect between the variables in question, the hypothesis was fully rejected. Given the predictive variables used in the model, maternal and paternal overcontrolling parenting does not explain a unique variance in the child’s anxiety (which was also the case in the general sample). Unlike the case in the general sample, the indirect associations between overcontrolling parenting and the child’s anxiety were not obtained for fathers and mothers separately, mainly due to differential effects on and of the mediators across parental gender. Specifically, the association between overcontrolling parenting and overprotection was significant only among fathers (*β* = 0.45, *p* < 0.001), while the associations between the mediators (parental overprotection, *β* = 0.24, *p* = 0.003, and locus of control, *β* = 0.38, *p* = 0.05) and child anxiety were significant only among mothers. Thus, the overall indirect paths between overcontrolling parenting and child anxiety resulted as insignificant for mothers and fathers, despite an indirect individual effect via maternal external locus of control, which was approaching significance at 5% (*β* = 0.24, *p* = 0.09). On the indirect effect level, therefore, the current hypothesis was fully rejected both for mothers and fathers, based on partially differential connection patterns with the mediating variables across gender.

## Discussion

The study’s central trajectories explaining child anxiety referred to the association between parent–child anxiety both directly and indirectly through parenting control variables (i.e., overcontrol, autonomy granting, and overprotection) and parental locus of control. Those hypothesized trajectories were confirmed in part. First and foremost, we found a strong-positive association between the parent’s and the child’s anxiety, whose direct effect existed above and beyond the accounted effects of all other parental variables. This unique association observed between parent–child anxiety is in accord with previous research findings directly linking parental and child anxiety both in clinical and non-clinical samples [33, 35–39, 62]. Importantly, since more research on parent and child anxiety was conducted with mothers than with fathers (e.g., [33, 34, 37, 39]), so far it is difficult to determine based on the research literature whether parental anxiety plays a differential etiological role in child’s anxiety across parental gender. The current findings demonstrated similar direct association pathways between parent and child anxiety across parental gender, suggesting that the mother’s and the father’s anxiety share a similar unique and distinct role in relation to child anxiety. These findings are partially inconsistent with the differential direct association found in previous research between parent and child anxiety across parental gender [62, 77]. Although this was not the case in the current study with regard to the indirect association observed between parent and child anxiety, as specified below, more research is required to clarify the etiological differentiation of mother’s and father’s anxiety in the context of child anxiety. On the theoretical level, our cross-sectional data exhibiting an association between parent and child anxiety may either indicate that parental anxiety positively affects the child’s anxiety, or the other way around. However, the large magnitude effects of unique associations observed here in this regard generally support the notion that anxiety tends to run intergenerationally in the family [9], consistent with the fact that anxious children are more likely to have a parent with anxiety problems than children without anxiety disorders [35, 78]. On a practical level, the robust evidence demonstrating a strong correlation between parental and child anxiety should affect the clinical strategies employed by child and family therapists. Specifically, clinicians would want to prioritize addressing parental anxiety as a pivotal intervention method for both preventing and treating anxiety disorders in children and adolescents. By incorporating this approach, therapeutic efforts can more effectively mitigate the intergenerational transmission of anxiety, thereby enhancing treatment outcomes and fostering healthier familial dynamics.

The major goal of the study was also to identify the mechanism that partially explains this relationship between parent–child anxiety. In this regard, the study’s findings identified parental overprotection as a distinct variable mediating this parent–child association among mothers, but not among fathers. Especially among mothers, a few previous works also identify parental control and anxiety belief as mechanisms mediating the association between parent–child anxiety [33, 46, 79], part of which have not found a direct link between the latter variables. Indeed, the information in the research literature regarding the link between parent and child anxiety is to a great extent more evident for mothers than for fathers [62]. This can derive from a few reasons, one of which was mentioned above, regarding the superiority in research studies conducted with mothers, generally focusing more on the maternal etiological role regarding child anxiety. Another explanation is theoretical in nature, referring to the differentiation between mothers and fathers in parental behaviors and communication, the involvement in the child’s education, and in time spent with the child in general [9, 80, 81]. Due to these factors, mothers possess greater chances than fathers of demonstrating heightened anxious cognitions and behaviors, thereby impacting how children perceive and contemplate their surrounding’s challenges and threats [62]. As for parental overprotection specifically, despite the literature depicting its substantial etiological role in child anxiety [19, 20, 22], so far not much research work has tested the specific role played by this pattern of parental control in the association between parent–child anxiety. In alignment with the few research studies in this regard [38], our finding is among the first to demonstrate the specific mediating mechanism played by maternal overprotection in the context of childhood anxiety, suggesting that children of anxious mothers tend to exhibit higher anxiety levels partially because their mothers are more overprotective. Perhaps maternal overprotection constitutes a manifestation of mothers’ general anxiety [25, 45], which makes this parental behavior a distinct mediator that explains the relationship between mother–child anxiety. That is to say, that parental overprotection per se rather than overcontrol, or lack of autonomy granting, is a distinct control practice uniquely linked to child anxiety, which corresponds to our premise regarding the differential role played by these parental control variables in the etiology of child anxiety. Maternal expressions of overprotection, particularly among mothers who themselves experience anxiety, should be a primary focus for clinicians working with anxious children. Clinicians should be particularly attentive to these dynamics, as maternal overprotection might exacerbate a child’s anxiety symptoms and hinder their ability to develop coping mechanisms. Furthermore, the differential impact of maternal versus paternal anxiety and behaviour on a child’s psychological adjustment necessitates a gender-sensitive approach also in clinical practice [62, 82]. Understanding these nuances can enhance intervention strategies, making them more effective in addressing the specific pathways through which maternal anxiety influences child anxiety. The necessity to discern between parental controlling variables conceptually and methodologically was not sufficiently addressed in the research literature on child’s anxiety. However, a very few previous studies’ results did demonstrate the differential role played by parental control variables, specifically overprotection and autonomy support, in relation to child anxiety, especially with respect to mothers (e.g., [21]). While our findings do not indicate whether maternal overprotection elicits children’s fearful responses and behaviors or vice versa, they substantially support the notion that this specific kind of parental controlling behavior, which is considerably more likely to be practiced by anxious mothers [22], might preserve child anxiety [25].

Interestingly, the father’s anxiety was associated with overcontrolling parenting practices (i.e., overcontrol and lack of autonomy granting) rather than with paternal overprotection, with none of these variables associated with the child’s anxiety. This further extends the evidence signifying the differential gender-contingent role in the parental etiology of children’s anxiety. Our findings suggest that the father’s and the mother’s anxiety are possibly reflected differently in their parental control behaviors that are related to the child’s anxiety. Indeed, consistent with more general literature about gender differences in parenting styles and practice [61, 83], our findings suggest that the mother’s anxiety elicits more overprotecting and over supporting parental practices, while the fathers’ anxiety elicits more limiting, overcontrolling, and even harsh practices. Perhaps this specific empirical information could be utilized to develop differential parental gender-based intervention designed to cope with a child’s anxiety and various other internalizing difficulties, especially in those cases when parental anxiety is identified as a factor playing a contributing role in this regard. However, none of the paternal control variables included in the model were uniquely associated in the current study with the child’s anxiety, leaving the mechanism explaining the association between the father’s and the child’s anxiety unclear. It looks like further research dealing with fathers’ anxiety will be required to test more theoretical trajectories for child anxiety, specifically designated for paternal parenting style. Indeed, the majority of theoretical models dealing with the parental etiology of children's anxiety fail to consider the differential ways in which mothers and fathers impact their children's anxiety [62].

In terms of parental gender-contingent role, the current study discovered that maternal locus of control may play a significant role in child anxiety, with their propensity to external attributions of the causes of their own and their children's problems [58] associated with their child’s anxiety. In our proposed model, parental locus of control was considerably associated with both maternal anxiety and parental controlling parenting, however it failed to mediate the former effects on the child’s anxiety. However, our finding linking external parental locus of control with child anxiety is in line with previous findings connecting this parental attributional variable with child’s mental health and emotional well-being [50, 57, 59, 60]. It is particularly valuable, given the very little recent empirical evidence that can be found in this specific etiological context (namely, parental locus of control and child anxiety). Since locus of control represents an attributional style and it is perceptual, rather than behavioral, in nature [52, 58], further work will need to inspect and clarify the mechanism explaining how the latter variable relates to, and presumably affects, child anxiety. Consistent with past research [52], we also found here that external locus of control in mothers is associated with elevated anxiety. Therefore, it is possible that mothers with external locus of control might be more emotionally expressive [50], which could contribute to increase the transmission of their anxiety to their children via several trajectories described in the introduction. Additionally, given some evidence indicating a correspondence in parent and child locus of control [56, 57], perhaps parental external locus of control shapes the child’s locus of control which, in turn, affects his/her anxiety level. While the current research model fell short of containing and examining this trajectory, further research can be used to extend its finding in this regard.

The study findings are limited in several ways. The most important one concerns to study’s data collection, which is based solely on parental reports regarding both the parent’s and the child’s variables. Aside fromthe risk of inflating the correlations between the study variables by using single informant [84], parental beliefs and reports on children’s anxiety specifically might be affected by the parent’s own anxiety [33, 79], resulting in possible biased assessments of the child’s anxiety level and false (or a least exaggerated) effect magnitudes of the associations between parent and child anxiety. Future research utilizing a similar model to explain the child’s anxiety should be conducted using multiple informant reports for the parental control variables and for child anxiety. Also, the study employed a scale with insufficient internal consistency reliability, the autonomy scale from the Parenting Style Inventory II (PSI-II; [69]), which may represent a methodological flaw and compromise the validity of the current findings. Future research aiming to replicate these findings should address this scale’s shortcomings, potentially considering more reliable measurement alternatives. Moreover, the direct and indirect effects of parental practices and parent anxiety embodied in the research model are based on cross-sectional data and should not be necessarily interpreted as causal. Specifically, the statistical relationship between parent and child anxiety both the direct and indirect associations can be also understood in the terms of the opposite direction specified in the model (e.g., the child’s anxiety elicits parental overprotection and/or external locus of control anxiety and, in turn, parental anxiety). In order to clarify the causality of these relationships, more sophisticated data collection methods must be employed.

## Summary

This study investigates the pathways linking parental and child anxiety, focusing on both direct and indirect associations through parenting control variables such as overcontrol, autonomy granting, and overprotection, alongside parental locus of control. The hypothesized trajectories were partially validated. A robust positive association was identified between parental and child anxiety, with this direct effect persisting even after accounting for other parental variables. The findings reveal similar direct pathways linking parent and child anxiety across both genders, indicating that both maternal and paternal anxiety play distinct yet significant roles in the child’s anxiety. However, the indirect pathways differed by parental gender. For mothers, the relationship between parent and child anxiety was partially mediated by maternal overprotection. Additionally, a maternal external locus of control was positively correlated with child anxiety, independent of other maternal factors. Maternal overprotection, therefore, especially among mothers experiencing anxiety and external locus of control, should be a focal point for clinicians addressing child anxiety. The differential effects of maternal versus paternal anxiety and controlling behaviors on a child’s psychological adjustment highlight the need for gender-sensitive approaches in clinical practice [62, 82]. Recognizing these nuances could improve intervention strategies by targeting the specific mechanisms through which maternal and paternal anxiety influence child anxiety. The findings suggest that maternal anxiety is more likely to manifest in overprotective and overly supportive behaviors, while paternal anxiety tends to result in limiting, overcontrolling, or even harsh practices. This empirical evidence can inform the development of gender-specific interventions aimed at mitigating child anxiety and related internalizing difficulties, particularly in cases where parental anxiety is identified as a contributing factor.

## Data Availability

No datasets were generated or analysed during the current study.
